# Semi-Hardwood Cutting Propagation of Autochthonous Olive Varieties from Galicia: A First Evaluation of Rooting Ability

**DOI:** 10.3390/plants15162506

**Published:** 2026-08-19

**Authors:** Adrián López-Villamor, Susana Boso, Pilar Gago, José-Luis Santiago, María-Carmen Martínez

**Affiliations:** Grupo VIOR, Misión Biológica de Galicia (Consejo Superior de Investigaciones Científicas—CSIC), Carballeira 8, Salcedo, 36143 Pontevedra, Spain; adrianvilamor@mbg.csic.es (A.L.-V.); susanab@mbg.csic.es (S.B.); pgago@mbg.csic.es (P.G.); santi@mbg.csic.es (J.-L.S.)

**Keywords:** *Olea europaea*, semi-hardwood cuttings, rooting, autochthonous varieties, vegetative propagation, *indole-3-butyric acid* (IBA)

## Abstract

Olive trees (*Olea europaea* L.) show propagation behaviour that is strongly dependent on genotype. A dozen previously unknown autochthonous olive varieties from Galicia (north-western Spain) have recently been identified through molecular and botanical characterisation techniques. However, their suitability for vegetative propagation remains unknown. Survival, callus formation and rooting were evaluated in semi-hardwood cuttings of 12 autochthonous Galician varieties and the control variety Cobrançosa in two trials carried out in winter–spring and summer-–autumn 2025 (676 and 622 cuttings, respectively). Binary variables were analysed using generalised linear models (binomial distribution, logit link). The variety effect was highly significant for all three variables (*p* < 0.0001), while the cutting date was not significant as a main effect; the variety × date interaction was significant for survival and rooting but not for callus formation. Mean rooting increased from 19% in winter to 61% in summer, although some varieties showed the opposite pattern. A strong negative correlation was found between flowering on the mother plant and rooting, and no correlation was found between callus formation and rooting. These results provide a first quantitative reference on the vegetative propagation of autochthonous Galician olive varieties and supply baseline information for their multiplication and conservation.

## 1. Introduction

*Olea europaea* L. is one of the most important woody crops of the Mediterranean basin, although it has also been traditionally cultivated in marginal and non-Mediterranean areas, such as Galicia (north-western Spain), where olive growing has a long historical presence [1,2]. Preserving the genetic identity of each variety requires clonal propagation, which is traditionally achieved by semi-hardwood cuttings. This technique is simpler and less costly than in vitro culture, but its success varies widely with genotype [3]: rooting percentages range from above 80–90% in easy-to-propagate cultivars to only 5–20% in strongly recalcitrant ones such as the Portuguese cultivar ‘Galega vulgar’ [4]. In addition to genotype, the timing of cutting collection also modulates rooting response in a cultivar-specific way through its effect on shoot lignification and carbohydrate content, as described for the Italian cultivars ‘Frantoio’ and ‘Gentile di Larino’ [5] and for the cultivar ‘Sevillano’ [6].

Galicia (north-western Spain) supported a substantial area of olive cultivation until the Middle Ages, when heavy taxation of olive trees and other economic and political factors [7] drove a rapid decline, and olive cultivation had virtually disappeared by the twentieth century. A recent survey of 117 centuries-old olive trees scattered across the region combined microsatellite (SSR) genotyping with full botanical characterisation following UPOV descriptors, leading to the identification and description of twelve previously unknown olive varieties [1,2], which are well adapted to the soil and climatic conditions of their region of origin and produce distinctive, high-quality fruits. None of the surveyed trees tested positive for the regulated pathogens considered under EU legislation (*Verticillium dahliae*, *Xylella fastidiosa*, *Pseudomonas savastanoi* pv. *savastanoi*, or the surveyed viruses), providing a phytosanitary green light for using this material as a source for vegetative propagation [2].

Transferring this heritage-recovery effort to the production sector—nurseries, growers and the authorities involved in variety registration and commercialisation—requires effective, variety-specific propagation protocols, because rooting behaviour cannot be extrapolated from one variety to another [3]. Gago et al. [2] reported having recently begun propagating this material by cuttings for future agronomic characterisation, explicitly identifying this as a necessary next step; the present work reports the results of that propagation effort. Concurrently, and within the framework of a collaborative research project involving the authors of the present article, in vitro micropropagation experiments were conducted on the same varieties using the same source plant material and demonstrated significant differences among genotypes in their response to this alternative propagation approach [8].

*Castanea sativa* Mill. and its hybrids show a similar genotype-dependent rooting response, with moderate heritability and a substantial contribution of non-additive genetic variance [9]. A comparable pattern, combining a marked cultivar effect with cultivar × environment interactions, could therefore be expected in olive. To date, no data have been published on the semi-hardwood cutting propagation ability of these autochthonous Galician olive varieties. This study provides a first quantitative evaluation of the following, under a single standard propagation protocol, as a basis for future more targeted optimisation of propagation protocols for these varieties (i) survival, callus formation, and rooting in semi-hardwood cuttings of 12 autochthonous Galician olive varieties and of a Portuguese control variety (Cobrançosa); (ii) the effect of variety, cutting date (winter vs. summer), and their interaction on these variables; and (iii) the relationships between survival, callus formation, flowering on the mother plant, and rooting.

## 2. Results

**Figure 1:**
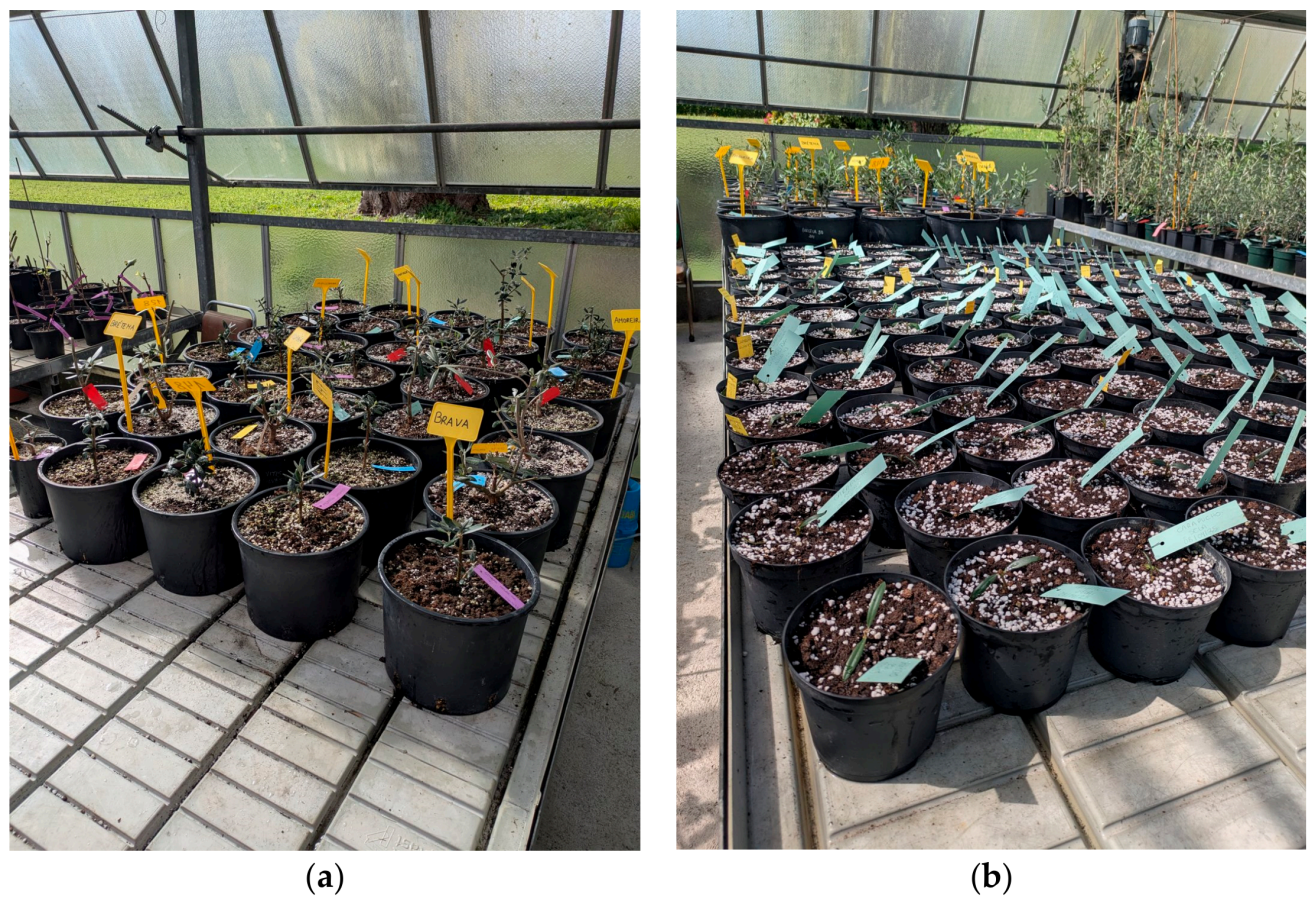
Representative view of the propagation trial in the VIOR group greenhouse. (**a**) Labelled mother plants of the autochthonous varieties (e.g., Brétema, Brava Gallega, and Amoreira) established in pots prior to cutting collection. (**b**) Cuttings from multiple varieties, individually labelled, at an early stage after transplanting.

**Figure 2:**
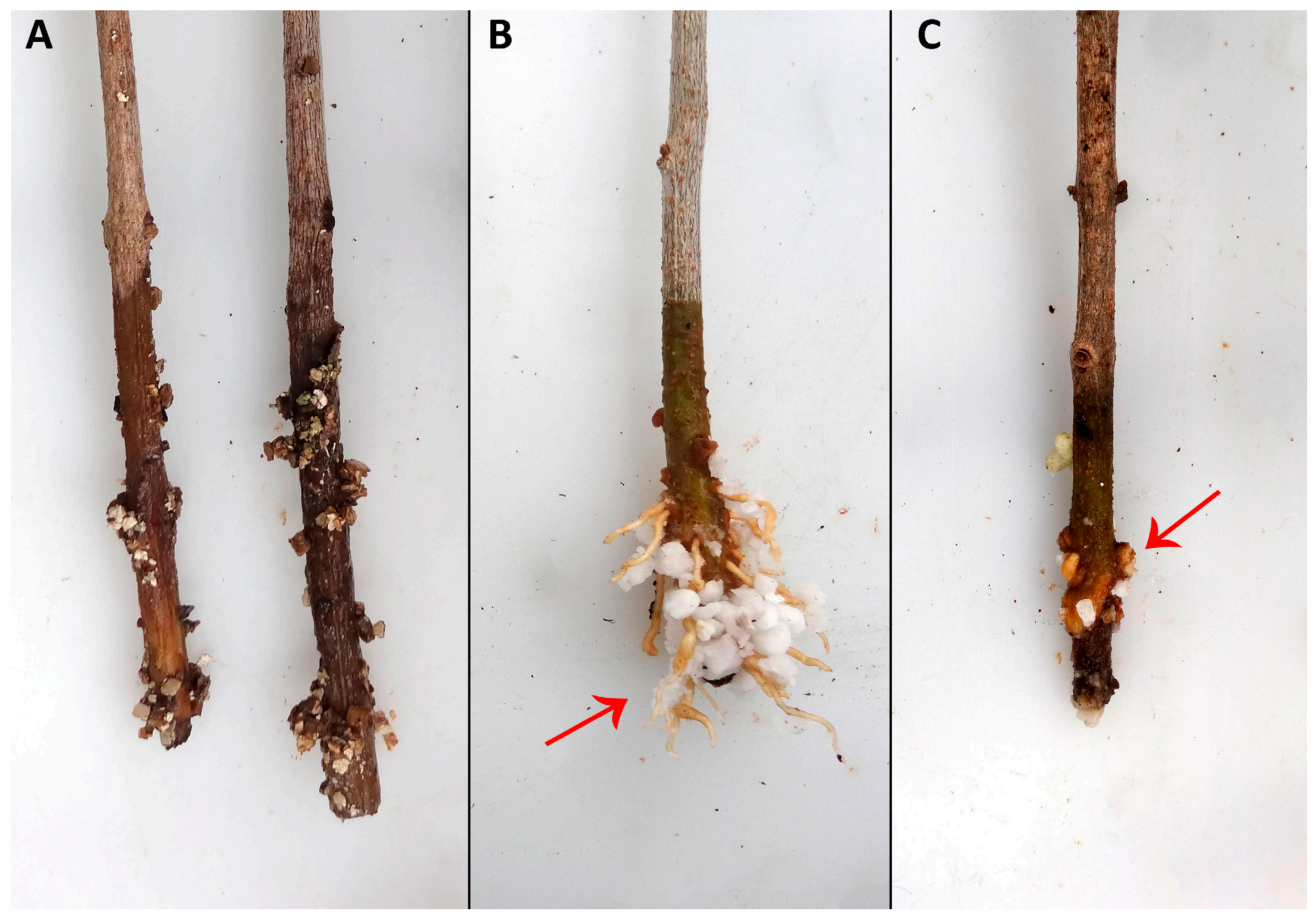
Representative responses of semi-hardwood olive cuttings at the end of the propagation trial. (**A**) Viable cutting showing neither callus formation nor adventitious root development. (**B**) Successful adventitious rooting with a well-developed root system (arrow). (**C**) Cutting showing callus formation at the basal end (arrow) in the absence of adventitious roots.

Figure 1 and Figure 2 show different stages of the propagation trials.

For the three variables analysed (survival, callus formation and rooting), the variety effect was highly significant, while cutting date was not significant in any case (Table 1). The variety × date interaction was significant for survival and rooting (*p* < 0.0001) but not for callus formation (*p* = 0.99). See the Section 3 for the likely statistical cause.

### 2.1. Winter Cuttings (F1)

**Figure 3:**
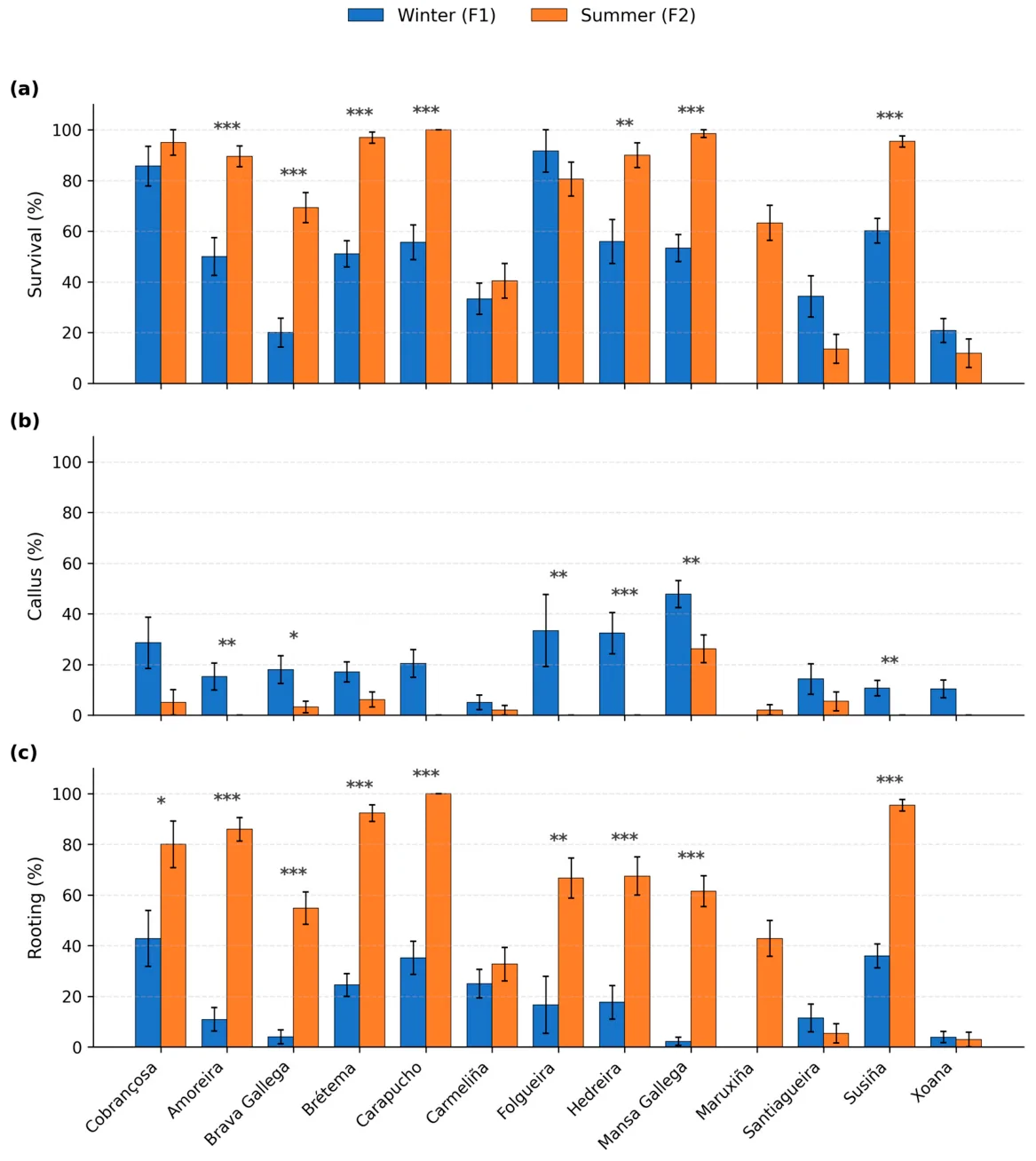
Survival, callus formation and rooting (%) by variety in the winter (F1) and summer (F2) trials. (**a**) Survival; (**b**) callus formation; (**c**) rooting. Asterisks indicate significant differences between the winter and summer trials within each variety (Fisher’s exact test on individual cuttings: * *p* < 0.05; ** *p* < 0.01; *** *p* < 0.001). These comparisons were performed separately for each variety and do not correspond to the overall variety × date interaction test reported in Table 1.

In the winter trial (2025), mean survival was 51%, callus formation 21%, and rooting 19% (Table 2 and Figure 3). Folgueira and Cobrançosa (control) showed the highest survival rates, at 92% and 86%, respectively. Cobrançosa also exhibited the highest rooting percentage (43%). Mansa Gallega showed the highest callus formation rate (48%) but the lowest rooting percentage (2% of cuttings), whereas Carmeliña showed the lowest callus formation rate (5%).

A positive correlation (0.55) was found between survival and rooting and a strong negative correlation between flowering on the mother plant and rooting (−0.75) (Table 3).

### 2.2. Summer Cuttings (F2)

In the summer trial (2025), mean survival reached 73%, with some varieties reaching 100% (Carapucho) and others showing very low values, such as Santiagueira and Xoana (14% and 12%, respectively) (Table 2 and Figure 3). Callus formation generally decreased compared with the winter trial, reaching 0 in many cases; Mansa Gallega again showed the highest percentage (26%), as in winter. Mean rooting increased from 19% in the winter trial to 61% in the summer trial, with some varieties close to 100% (Carapucho, 100%; Susiña, 95%). A very strong positive correlation was found between survival and rooting (0.83). Rooting in the winter and summer trials was also positively correlated (ρ = 0.59), suggesting a moderate consistency of varietal rooting performance across seasons (Table 3).

### 2.3. Correlations Between Cutting Dates

Spearman correlations between the variables recorded on both dates are shown in Table 3. In addition to the correlations already noted, a positive and significant association between survival and callus formation on both dates (0.58 and 0.59) stands out, together with the absence of a significant correlation between callus formation and rooting on either date.

Flowering in the winter trial was an all-or-nothing, variety-level trait rather than a graded one: 100% of cuttings scored positive for flowering (floweringF1 = 1) in exactly three varieties—Brava Gallega, Mansa Gallega and Xoana—and 0% in the remaining ten, including the control. All three flowering varieties showed very low winter rooting (2–4%). The −0.75 correlation above therefore reflects a contrast between these three varieties and the rest, rather than a continuous, within-variety dose–response relationship, and should be interpreted with this caveat in mind.

## 3. Discussion

The variety effect was, by far, the most determinant factor for survival, callus formation and rooting (Table 1), confirming that semi-hardwood cutting behaviour in olive is markedly variety-dependent. These results are consistent with those reported in other olive varietal collections [4,5] and with observations made by other authors in cultivars of other agriculturally important and recalcitrant woody species, such as chestnut (*Castanea sativa* Mill. and its hybrids), where the rooting ability of cuttings also showed strong genetic dependence [9]. Among the Galician autochthonous varieties, Folgueira and the control variety Cobrançosa stood out for their ease of propagation in winter, and Carapucho, Susiña, Brétema and Mansa Gallega in summer, while Xoana and Santiagueira behaved as recalcitrant on both dates, with rooting percentages comparable to those described for the Portuguese cultivar ‘Galega vulgar’ (5–20%) [4]. Before discarding these latter varieties as difficult to propagate, it would be worth testing specific treatments (IBA concentration, substrate, mother-plant age or vigour), since recalcitrance to cutting propagation can be partially mitigated through protocol adjustments [4]. The marked variety effect detected in this study is consistent with the molecular and botanical characterisation of this material: Gago et al. [2] confirmed, using microsatellite markers and UPOV descriptors, that the autochthonous Galician olive varieties constitute genetically distinct resources, both from each other and from reference Mediterranean cultivars, rather than clones or synonyms of already known varieties. This genuine genetic diversity, shaped by centuries of adaptation to Galicia’s environmental conditions, provides a direct explanation for the wide range of behaviours observed here, from easily propagated to clearly recalcitrant varieties, and reinforces the need for variety-specific propagation protocols rather than a single protocol for the whole germplasm collection.

Although cutting date showed no significant main effect overall, the variety × date interaction was significant for survival and rooting, indicating that the effect of timing on these two variables depends on genotype, whereas no significant variety × date interaction was detected for callus formation. This is best explained statistically rather than biologically: the callus formation model showed quasi-complete separation in several genotype × date cells with 0% or 100% observed callus (e.g., Amoreira F2, Carapucho F2, Folgueira F2, and Hedreira F2), consistent with the sparse, often near-binary callus response observed across varieties (Table 2); this reduces the estimable degrees of freedom for the interaction term and destabilises the corresponding F-test (see Section 4.3). In most varieties, rooting increased markedly from the winter to the summer trial (e.g., Carapucho, Susiña, Amoreira or Mansa Gallega), a pattern consistent with that described by [5] for ‘Frantoio’ and ‘Gentile di Larino’, in which the timing of material collection—and the degree of lignification and carbohydrate reserves of the shoot at that time—modulates rooting response differently depending on the cultivar. However, Santiagueira and Xoana showed the opposite pattern (better performance in winter than in summer), reinforcing the idea that there is no single optimal propagation window valid for all varieties. A plausible physiological explanation for this genotype-dependent seasonal response is that winter and summer cuttings differ in their physiological status, including endogenous auxin levels, carbohydrate reserves and the degree of shoot lignification, all factors known to influence adventitious root formation in olive [10], and that varieties differ in how strongly each of these factors constrains rooting at a given time of year; testing this hypothesis directly—for example through carbohydrate or hormone profiling of the shoots at collection—is a priority for future work. From a practical standpoint, these results suggest that nurseries should prioritise summer collection for most of these varieties but winter collection specifically for Santiagueira and Xoana.

The strong negative correlation observed between the presence of flowering on the mother plant and cutting rooting (ρ = −0.75) is consistent with the well-known competition between reproductive development and rhizogenesis for the carbohydrate reserves available in the mother plant and in the cutting itself [3]. As detailed in Section 2.3, flowering in the winter trial was an all-or-nothing, variety-level trait, present in three varieties (Brava Gallega, Mansa Gallega and Xoana) and absent in the remaining ten, with all three flowering varieties also showing very low winter rooting (2–4%); the correlation therefore reflects a contrast between these three varieties and the rest rather than a continuous, within-variety relationship. Nonetheless, it directly suggests a practical intervention: removing flower buds (disbudding) from mother plants of varieties prone to flowering to maximise rooting performance in subsequent cutting cycles.

Finally, the absence of a significant correlation between callus formation and rooting on either date indicates that, under the conditions of this study, a callus is neither an obligatory step nor a good predictor of subsequent rooting. This agrees with the review of Porfírio et al. [10], who reported that in olive, callogenesis and adventitious root formation are related but partly independent developmental processes. Although both responses may be induced by auxin treatment and wound healing, adventitious roots do not necessarily arise from callus tissue, and abundant callus formation is not consistently associated with improved rooting performance. Callus formation and rooting appear to be governed by at least partially independent processes in these varieties, which may originate through distinct cellular differentiation pathways in recalcitrant woody species. This has a direct practical implication: the presence of a callus should not be used as an early selection criterion for varieties or cuttings with good rooting potential. It should also be noted that callus was scored here as a binary presence/absence trait; a quantitative or ordinal scale (e.g., callus size or coverage) would allow a more nuanced test of its relationship with rooting in future work.

Genotype-dependent recalcitrance to cutting propagation is not unique to olive. In chestnut (*Castanea sativa* Mill. and its hybrids), another rooting-recalcitrant woody species, a previous study [9] found that rooting ability under optimised juvenile conditions can reach very high values (91% and 82% in two trials), far above the 64% mean obtained from 60-year-old mother plants of the same genotypes, illustrating how strongly maturation state constrains rooting independently of genotype. That same study showed rooting ability to be under moderate genetic control (broad-sense heritability Ĥ^2^ = 0.33–0.43), driven mainly by dominance and epistasis rather than additive effects (narrow-sense heritability < 0.10 in all cases), so that selecting for rooting ability had to rely on choosing the best-rooting individual clones or families rather than on general parental breeding values [9]. The present olive study cannot partition genetic variance into additive and non-additive components since it compares named varieties rather than structured full-sib families; nonetheless, the wide range of behaviours found here, from Cobrançosa and Folgueira (easy-to-root) to Xoana and Santiagueira (recalcitrant), is consistent with genotype-level control of comparable magnitude, and a controlled-cross breeding design, as used in chestnut, would be a logical next step for exploring the genetic architecture of rooting in these Galician varieties. Beyond chestnut, similar genotype-dependent recalcitrance to cutting propagation has been reported in walnut (*Juglans* spp.), where the rooting of cuttings is notoriously difficult and highly genotype-dependent, to the point that clonal propagation of many valuable genotypes remains largely unresolved despite decades of work [11], and in grapevine (*Vitis* spp.), where a recent QTL study on a large rootstock mapping population found that rooting success in hardwood cuttings has a polygenic genetic architecture, with individual loci explaining only 3–4% of the phenotypic variance, and that the genetic determinants differ between hardwood cuttings and grafted plants [12]. Together with the present results, this reinforces the view that cutting propagation ability in woody perennials is typically governed by many factors of small individual effect and by genotype-specific physiological constraints, rather than by a single, easily selectable trait—supporting the case for variety-specific rather than universal propagation protocols.

This study has several limitations that should guide the interpretation of its results and the design of future work. A single IBA concentration (2 g/L) and no alternative auxin were tested, so genotype differences in rooting cannot be fully disentangled from a possibly suboptimal hormone treatment for some varieties; concentration and auxin-type trials are a priority, particularly for the more recalcitrant varieties (Xoana and Santiagueira). The winter–summer contrast reported here reflects a single annual cycle and a single mother plant per variety, grown under ambient, non-continuously monitored greenhouse conditions; confirming these patterns over successive years, with more than one mother plant per variety and logged environmental data, would strengthen their generality. Rooting and callus formation were recorded as binary (presence/absence) traits, and no root-quality metrics (root number, length, and biomass) or post-transplant survival were measured; quantitative root-quality assessment is needed before translating these results into definitive nursery recommendations. Finally, rooting behaviour was characterised here at the phenotypic level only; candidate-gene or QTL-level analyses, such as those reported for the AOX1 subfamily during IBA-induced adventitious rooting in ‘Galega vulgar’ [4], were outside the scope of this work and are a natural next step.

Despite these limitations, this study provides the first quantitative reference on the vegetative propagation ability of autochthonous Galician olive varieties identified to date, supplying information that is needed both by the nurseries collaborating in their multiplication and for the conservation and registration goals pursued by the administrations and entities involved. The work also fulfils a need previously identified by Gago et al. [2], who reported having just begun cutting-based propagation of this material for future agronomic characterisation; this study can be regarded as reporting the first results of that effort. Furthermore, the results presented here are complementary to those being obtained in parallel, within the same Operational Group, for in vitro micropropagation of these varieties [8]. Together, both approaches will contribute to the development of variety-specific multiplication protocols and help identify the most efficient propagation strategy for each genotype.

## 4. Materials and Methods

### 4.1. Plant Material

The plants used in the rooting trials were 12 autochthonous Galician olive varieties (*Olea europaea* L.) identified and characterised botanically and molecularly by the VIOR Research Group in the Misión Biológica de Galicia institute (MBG-CSIC) (Table 4). In September 2024, three rooted cuttings, one or two years old, established in 1 L pots of substrate (peat:perlite, 2:1), were selected from each of the 12 Galician varieties plus one plant of the control variety Cobrançosa (of Portuguese origin) and transplanted into 5 L pots (same substrate) to obtain the mother plants used in the vegetative propagation trials by semi-hardwood cuttings. A single mother plant was used per variety; this is acknowledged as a limitation in Section 3. These plants were kept in the VIOR group’s greenhouse under natural, non-continuously logged temperature, light and humidity conditions, with one or two waterings per week depending on the season.

To improve the semi-hardwood cutting propagation system for these varieties and to study their rooting ability, two trials were carried out: the first in winter–spring 2025 (18 February to 8 April) and the second in summer–autumn of the same year (12 August to 16 October). In the first trial, cuttings were obtained from 11 of the 12 varieties plus the control variety, with an average of 56 ± 29 cuttings per variety and 676 cuttings in total (Table 4). Pruning of the mother plants, together with fertilisation with Multicote 6 (15-7-15 + 1MgO + microelements, controlled-release fertiliser) at a dose of 5 g per plant, induced the emission of new shoots from adventitious buds at the base; these shoots, formed from a mix of normal and adventitious buds, were used to obtain the cuttings for the second trial, which included all 12 autochthonous varieties plus the control variety, with an average of 48 ± 20 cuttings per variety and 622 in total (Table 4). Mother plants were pruned at the time of each cutting collection (February and August 2025), and the fertiliser was applied at the end of March 2025 after the first collection; only shoots arising from adventitious buds at the base of the plants were used as cutting material.

### 4.2. Cutting Conditions

Shoots were cut obliquely above and below the buds to obtain cuttings with 4 buds and two terminal leaves at the top. Cuttings were immersed for 2 min in an aqueous solution with a systemic fungicide (azoxystrobin + difenoconazole; Ortiva^®^ Top, Syngenta, Basel, Switzerland) at 1 mL/L and then treated for 3 min with an aqueous solution of indole-3-butyric acid (IBA) (INABAR) at a concentration of 2 g/L; a single IBA concentration and no alternative auxin were tested (see Section 3, Discussion). They were then planted in 50-cell trays, with 10 g of peat:perlite substrate (2:1) per cell. Both trials were carried out in a growth chamber with 16 h light at 25 °C and 8 h dark at 20 °C, 90% relative humidity, and fog-system irrigation every 15 min (≈15 s duration); these conditions were maintained for the first month. During the second month, humidity was progressively reduced to 50% and daytime temperature increased to 28 °C to promote acclimatisation of the new plants before transplanting to the VIOR group’s greenhouses at the end of each trial.

At the time of transplanting, the binary variables survival (1: alive; 0: dead), callus (1: callus present; 0: absent) and rooting (1: root present; 0: absent) (see Figure 2) were recorded for each cutting. In the first trial, since flowering was observed in some varieties, the variable flowering on the mother plant (floweringF1: 1: present; 0: absent) was also recorded. Each cutting was transplanted into a 1 L pot with the same substrate used in the rooting trials labelled with the variety name and transplant date.

### 4.3. Statistical Analysis

The binary variables recorded on the cuttings (survival, callus formation and rooting) were analysed independently using generalised linear models with a binomial distribution and logit link and fitted in PROC GLIMMIX (SAS v.9.4, SAS Institute Inc., Cary, NC, USA) [13], with variety, cutting date and their interaction (variety × date) as fixed effects; no random effect was included, given that a single mother plant per variety was used. Adjusted means (LSMeans) were back-transformed to the probability scale via the inverse logit link (ILINK option); no adjustment for multiple comparisons was applied, as only individual-variety LSMeans were estimated (no pairwise comparisons were requested). Fixed effects were tested with F-tests, using the conventional thresholds *p* < 0.05, *p* < 0.01 and *p* < 0.001. Goodness-of-fit was adequate for all three models, with a Pearson Chi-Square/DF of 1.01 (survival), 0.81 (callus) and 1.01 (rooting), indicating no meaningful overdispersion; the callus model nonetheless showed quasi-complete separation in several genotype × date cells with 0% or 100% observed values, which is reflected in the non-significant variety × date interaction reported for that variable (Table 1; see Section 3). In addition, the difference between the two cutting dates within each variety (Figure 3) was tested separately using Fisher’s exact test on the individual cuttings.

To assess the consistency between the two cutting dates, non-parametric Spearman correlations (ρ) were calculated between the binary variables recorded on each cutting (survival, callus, rooting and, where applicable, flowering on the mother plant), because these variables are dichotomous (0/1). Data were first summarised as proportions per genotype × date then reorganised into wide format (one pair of values per variable, Date 1 and Date 2, for each variety), and correlations were calculated using PROC CORR (SAS Institute Inc., Cary, NC, USA) [13]. Correlations with *p* < 0.05 were considered significant.

## 5. Conclusions

Semi-hardwood cutting propagation proved a viable technique for the vegetative multiplication of these autochthonous Galician olive varieties, although rooting success was markedly genotype-dependent and no single cutting season was optimal for all varieties. Flowering on the mother plant reduced subsequent rooting, whereas callus formation was not a useful predictor of it. These results provide a first quantitative reference for the multiplication and conservation of this germplasm and should be validated over successive seasons with additional mother plants and plant material of different origin.

## Figures and Tables

**Table 1:** Effect of variety (12 autochthonous Galician olive varieties + control variety Cobrançosa), cutting date (winter/summer) and their interaction on survival, callus formation and rooting (PROC GLIMMIX, SAS v.9.4). Values are based on a confirmatory re-analysis of the final dataset (Section 4.3).

Variable	Effect	F Value	*p* Value
Survival	Variety	15.55	<0.0001
	Date	0.01	0.9378
	Variety × Date	6.10	<0.0001
Callus formation	Variety	4.94	<0.0001
	Date	0.00	0.9651
	Variety × Date	0.20	0.9944
Rooting	Variety	11.37	<0.0001
	Date	0.01	0.9127
	Variety × Date	6.01	<0.0001

**Table 2:** Survival, callus formation and rooting (%) of cuttings for each Galician variety and the control variety in the winter (F1) and summer (F2) trials. Cell shading indicates relative value (red, lowest, to dark green, highest; see legend below the table).

Variety	Survival %		Callus %		Rooting %	
	F1	F2	F1	F2	F1	F2
Cobrançosa (Control)	86%	95%	29%	5%	43%	80%
Amoreira	50%	89%	15%	0%	11%	86%
Brava Gallega	20%	69%	18%	3%	4%	55%
Brétema	51%	97%	17%	6%	24%	92%
Carapucho	56%	100%	20%	0%	35%	100%
Carmeliña	33%	40%	5%	2%	25%	33%
Folgueira	92%	81%	33%	0%	17%	67%
Hedreira	56%	90%	32%	0%	18%	68%
Mansa Gallega	53%	98%	48%	26%	2%	62%
Maruxiña	N/A	63%	N/A	2%	N/A	43%
Santiagueira	34%	14%	14%	5%	11%	5%
Susiña	60%	95%	11%	0%	36%	95%
Xoana	21%	12%	10%	0%	4%	3%
**Mean**	51%	73%	21%	4%	19%	61%
	0–19%	20–39%	40–59%	60–79%	80–89%	90–100%

**Table 3:** Non-parametric Spearman correlations between the binary variables survival, callus formation and rooting (winter, F1; summer, F2), including flowering on the mother plant (floweringF1). Coefficients marked with * are significant (*p* < 0.05). PROC CORR, SAS v.9.4.

Variable	Survival F1	Survival F2	Callus F1	Callus F2	Flowering F1	Rooting F1	Rooting F2
SurvivalF1	1	0.68 *	0.58 *	−0.19	−0.53	0.55 *	0.69 *
Survival F2		1	0.59 *	−0.08	−0.2	0.37	0.83 *
Callus F1			1	0.18	0.08	−0.11	0.33
Callus F2				1	0.24	−0.16	−0.42
Flowering F1					1	−0.75 *	−0.53
Rooting F1						1	0.59 *
Rooting F2							1

**Table 4:** Number of cuttings of each variety used in each of the two trials (winter, F1; summer, F2).

Variety	Winter (F1)	Summer (F2)
Cobrançosa (Control)	21	20
Amoreira	46	57
Brava Gallega	50	62
Brétema	94	65
Carapucho	54	18
Carmeliña	60	52
Folgueira	12	36
Hedreira	34	40
Mansa Gallega	90	65
Maruxiña	0	49
Santiagueira	35	37
Susiña	103	87
Xoana	77	34
**Total**	**676**	**622**

## Data Availability

The data presented in this study are available on request from the corresponding author. The data are not publicly available due to privacy and ethical restrictions.
